# Psychometric Testing of the Korean Version of the Attitudes toward the Advance Directives in Low-Income Chronically Ill Older Adults

**DOI:** 10.3390/healthcare8010062

**Published:** 2020-03-18

**Authors:** JinShil Kim, Seongkum Heo, Sun Woo Hong, HeeRyang Kim, Ahrang Jung, Minjeong An, JaeLan Shim

**Affiliations:** 1 College of Nursing, Gachon University, Incheon 21936, Korea; blushil68@gmail.com; 2 Georgia Baptist College of Nursing, Mercer University, Atlanta, GA 30341, USA; Heo_s@mercer.edu; 3 Department of Emergency Medical Services, Daejeon University, Daejeon 34520, Korea; swhong@dju.kr; 4 Gwangju City Public Health Center, Gwangju 12738, Korea; msg86@daum.net; 5 School of Nursing, University of North Carolina, Chapel Hill, NC 27514, USA; ajung@email.unc.edu; 6 College of Nursing, Chonnam National University, Gwangju 61469, Korea; 7 Department of Nursing, Dongguk University, Gyeongju 38066, Korea

**Keywords:** advance directives, attitude, factor analysis, psychometrics, older adults, poor, chronic diseases, community

## Abstract

The purpose of this study was to examine the psychometric properties of the Advance Directive Attitude Survey in Korean (K-ADAS), a measure of attitudes toward advance directives (ADs). A total of 118 low-income, community-dwelling older adults (mean age, 75.09 years) participated. An exploratory factor analysis (EFA) was conducted to determine the factor structure of the K-ADAS. Validity was further assessed by known associations of the K-ADAS with perceived susceptibility and severity using part of the Advance Care Planning surveys. Its reliability was examined by calculating alpha coefficients. EFA determined a three-factor structure model with good model fit. Validity was further supported with significant correlations between the K-ADAS and susceptibility and severity. Reliability was supported by adequate level of Cronbach’s alpha. The K-ADAS was a valid and reliable measure for assessment of AD attitudes with a sound model fit. Thus, the K-ADAS can be used to assess AD attitudes among community-dwelling elders.

## 1. Introduction

Longer lives with multiple chronic conditions are becoming increasingly burdensome to elderly people, as well as current society [1,2,3,4]. In the United States, based on the National Health Interview Survey in 2012, more than half of American adults were estimated to have one or more chronic conditions [5]. According to a recent report, most older Koreans aged 65 years or older (88.5%) had one or more chronic health conditions with an average of 2.5 multi-morbidities [6]. Furthermore, the prevalence of such major chronic diseases in Korean adults is projected to increase approximately 1.6- to 2.5-fold by 2040 [7]. Despite improvements in controlling risk factors of these morbidities and healthcare progress [8], the likelihood of living with these multi-morbidities largely increases with age [9,10,11]. 

Living with multi-morbidities often involves considerable physical, psychological, and financial impact from associated emergency department visits or hospitalizations for treatment, therapeutic procedures, and unexpected adversities or avoidable high mortality [4,12]. These chronically ill people and/or their families who may or may not have life-limiting conditions are often exposed to a spectrum of decision-making situations for medical care, including therapeutic or screening options, or future end-of-life (EOL) care. Advance directives (ADs) specify an individual’s treatment preference in the future, and they can be used from being exclusively available to those patients with terminal conditions to general populations who may or may not have chronic diseases to make such decisions ahead of time [13,14]. Thus, preparing ADs during advance care planning (ACP) is highly recommended in Western countries, not only for patients with cancer or terminal illness but also for individuals with various diseases, such as heart failure or chronic obstructive pulmonary disease [15,16,17].

Use of ADs or ACP can result in several positive health outcomes, including less use of aggressive treatments, more patient and caregiver satisfaction, and reduced unnecessary costs [18,19]. Despite the recommendations for ACP or written ADs in malignancy and nonmalignancy settings and their positive health outcomes, access to ADs is still considerably limited to the public or nonmalignancy contexts [20,21,22,23] and people with low financial status [24,25,26]. For instance, only approximately 26% of American adults and only 12.7% of admitted patients with heart failure have documented ADs [22,27]. Approximately 41% of community-dwelling elderly had both a health care proxy and a living will [28]. A lack of or limited access to ACP and/or ADs led to poor quality of care among the financially vulnerable older adults [24,25,26]. Thus, use of ADs need to be improved. The first step is to assess factors affecting ADs using reliable and valid instruments.

One of the barriers to the limited access to or suboptimal use of ADs could be negative attitudes toward ADs, which may lead to reluctance to engage in discussion about disease progression and/or decisions for end-of-life care [29,30]. Thus, exploring people’s attitudes toward such care using reliable and valid measures could be helpful to facilitate and increase earlier and extended use of ADs in clinical and nonclinical contexts. The Advance Directives Attitude Survey (ADAS) is one measure that evaluates attitudes toward ADs and decisions for EOL treatment and was used by the developers [31] and by Lee et al. [32,33] among community-dwelling older Koreans and older cancer patients after the version of the ADAS (16 items) was translated into Korean. However, no full psychometric property tests, including reliability and construct validity tests, were performed for the original English version or the Korean version of the ADAS. It is critical to use a reliable and valid instrument to assess a phenomenon appropriately. Thus, the psychometric properties of the ADAS need to be tested.

When we test psychometric properties of an instrument, we have to consider the socio-cultural circumstances. Income levels have been associated with use of ADs and ACP. For example, use of ADs and ACP was poorer in cancer patients with low incomes compared to that in those with high incomes [34]. The level of knowledge regarding ADs and ACP in community-dwelling older adults with low incomes was also lower than that in those with high incomes [26], which may affect completion of ADs or EOL discussion [35]. Culture has also been associated with individuals’ EOL values and preferences, such as attitudes toward ADs [36,37,38]. Culture may also affect completion of ADs or EOL discussion [35,39], such as perceived severity and susceptibility [35]. Therefore, the psychometric properties of the Korean version of the ADAS need to be assessed in Korean patients with low incomes, considering relationships of perceived susceptibility and severity to AD attitudes. The specific objectives were to (1) test the construct validity of the K-ADAS using an exploratory factor analysis (EFA; Mplus 7.4) [40], (2) test the construct validity further by examining associations of perceived susceptibility and severity regarding adverse EOL experiences with the attitudes toward ADs, and (3) test the internal consistency reliability using Cronbach’s alpha in low-income chronically ill older adults in the community. 

## 2. Methods

### 2.1. Design and Procedure 

A cross-sectional study design was used to test the psychometric properties of the K-ADAS in a convenient sample of low-income, community-dwelling older adults with chronic diseases. Elderly home residents who receive a home visiting service for chronic disease management from a public health center participated in this study. The institutional review board of the university approved this study (ethical approval codes: 144396-201610-HR-077-01). A signed written informed consent statement was obtained from all participants who agreed to participate. Then, home visiting nurses conducted face-to-face interviews to collect data on attitudes toward ADs and perceived susceptibility and severity regarding adverse EOL experiences during their visitations.

### 2.2. Participants

Eligible individuals were (1) community-dwelling older adults 60 years old or older; (2) recipients of a home visit service for the management of chronic diseases (e.g., hypertension, diabetes, or cardiovascular diseases) and qualified by low income (bottom quintile of either family income or the payment of health insurance) [41]; and (3) those with capacity to understand the study protocol. Exclusion criteria were (a) a diagnosis of terminal conditions, such as terminal cancer or end-stage organ failures, chronic obstructive pulmonary disease, or heart failure; (b) current reception of palliative and/or hospice care; and (c) a serious cognitive impairment or personality change associated with neuropsychological disorders, such as Alzheimer’s disease, dementia, (traumatic) brain disorders, or mental disorders based on the information provided by the home visiting nurses. 

### 2.3. Measures

Advance directives attitude. Attitudes toward AD and decisions for EOL care were measured using the K-ADAS. The ADAS, which was originally developed as a 17-item measure [31], was revised as a 16-item questionnaire by M. Nolan© (2003). The scale consists of four components, which measure the extent of one’s positive or negative views about decisions regarding ADs, specifically (a) the opportunity for treatment choices, (b) impact of ADs on the family, (c) effects of ADs on treatment, and (d) illness perceptions (severity of their illness). The factor structures of the original or 16-item scale were not tested. Scores on the 16-item version are constructed on a four-point Likert scale (1 = strongly disagree and 4 = strongly agree). Possible scores range from 16 to 64, and higher scores indicate more positive attitudes toward ADs. Before calculation of the total score, negatively stated items (Items No. 7, 9, and 16) were reverse-coded, so that higher scores indicate more positive attitudes. The reliability of this instrument was supported by an alpha coefficient of 0.74 [31]. 

After obtaining permission to translate the ADAS into Korean from the author who possessed the copyright, a standard procedure was followed to develop the K-ADAS and to ensure the appropriate translation processes [42]. A total of seven experts were involved in translating, back-translating, evaluating the original and translated versions, and finalizing the translated version considering the comments from the experts and the semantic agreement and cultural adequacy. Two nurse scholars who had expertise in nursing research and were fluent in both English and Korean translated the scale independently and generated the draft versions of the K-ADAS. The extent of agreement between the two translators was then determined by a graduate nursing student using a four-point scale (1 “strong disagreement” to 4 “strong agreement”). Three nurse scholars then evaluated the translated version, regarding conceptual agreement and clarity, using a four-point scale (1 “strong disagreement” to 4 “strong agreement”) and reading level. The version with two-translator consent was then back-translated into English by a qualified translator who was proficient in English and Korean with a major in English education. Semantic agreement and/or idiomatic integrity was tested using three methods. First, the principal investigator who is fluent both English and Korean compared the back-translated version with the original questionnaire using a five-point scale (1 “strong disagreement” to 5 “strong agreement”). Second, an English native speaker compared the back-translated version with the original questionnaire using a four-point scale (1 “strongly disagreement” to 4 “strongly agreement”). Third, a Korean expert who had majored in Korean language education reviewed the translated Korean version to check the semantic and/or idiomatic integrity. Ambiguous words or phrases were reworded or rephrased considering cultural meaning and adequacy. For example, in Korean language, unlike English, honorific language should be used for research subjects. Thus, the term “you”, “treatments at the end of life”, and “have an AD” had to be translated into an honorific language. 

#### 2.3.1. Perceived Susceptibility and Severity

The Perceived Susceptibility/Severity Scale, a part of the Advance Care Planning Survey [35,43,44], is a measure assessing the extent of an individual’s perceived susceptibility and severity regarding end-of-life experiences and adverse consequences, given ACP or not, associated with unwanted treatment, costs, or family conflict. Both perceived susceptibility and severity subscales have five items each on a seven-point Likert response scale. Possible scores on perceived susceptibility and severity each range from 5 to 35, with higher scores indicating higher levels of perceived importance and concerns about the EOL experiences, respectively. The reliability was documented as desirable with Cronbach’s alphas of 0.73 for the perceived susceptibility and 0.76 for the perceived severity scales [35]. In this sample, Cronbach’s alphas for the scales were 0.88 and 0.84, respectively.

#### 2.3.2. Comorbidity and Demographic Characteristics

The comorbid conditions were measured by the Charlson comorbidity index [45]. A weighted value was assigned to each condition, with a higher sum of these weighted values indicating more comorbidities. Demographic data were collected using a standard form developed by the investigators, including age, gender, marital status, religious affiliation, types of chronic conditions, and experience of ADs.

### 2.4. Statistical Analysis

Data analyses were performed using the Statistical Package for Social Science Program, version 23.0 [46]. Descriptive statistics, including frequencies, percentages, means, and standard deviations, were used to describe the sample characteristics and variables. Item analyses were performed to assess the quality of the items (e.g., item means) [47]. The level of significance was set at a *p*-value of 0.05.

Construct validity related to the underlying conceptual structure of the instrument was tested through EFA using Mplus 7.4 [40]. Eigenvalues >1 were used to consider factor extractions, and the ProMax approach was used for rotation. To evaluate model fit of the K-ADAS, the comparative fit index (CFI), Tucker-Lewis index (TLI), root mean square error of approximation (RMSEA), and standardized root mean square residual (SRMR) were used. Final acceptance or rejection of models for the K-ADAS was determined by (1) conventional criteria for goodness of model fit: CFI ≥ 0.90, TLI ≥ 0.90, RMSEA ≤ 0.08, and SRMR ≤ 0.05 [48]; (2) factor loadings ≥ 0.45 as a cut-off point [49]; and (3) smaller values of Bayesian information criteria to identify model parsimony. Construct validity of the K-ADAS was further tested using Pearson’s correlation to examine the inter-correlation with perceived susceptibility and perceived severity. 

The internal consistency reliability of the K-ADAS was then assessed by calculating the Cronbach’s alpha coefficient and the item homogeneity of the instrument using item-to-total correlation. An acceptable coefficient for Cronbach’s alpha was ≥0.70 [47], and that for the item-total correlations was >0.30 [50].

## 3. Results

One hundred and eighteen older adults who had chronic illnesses residing in community settings participated in this study. The mean age of the sample was 75.09 (±7.21) years (range 60–90 years) (Table 1). The majority of the participants were female (83.1%) and had finished elementary school or lower (72.0%), and 39.3% were married. Two-thirds reported religious affiliations (66.1%). The mean score on the K-ADAS-16 was 46.49 ± 5.24 (Table 1).

### 3.1. Initial Psychometric Properties of the K-ADAS

We tested the construct validity and reliability of the K-ADAS (16 items) version first. 

#### 3.1.1. Construct Validity

Using EFA with maximum-likelihood estimation and ProMax rotation, dimensionality of the factor structure was specified from three to four factors based on the four components of the original ADAS, although the developers did not test the factor structure. Both the three- and four-factor solutions provided significantly acceptable fits of three- and four-factor solutions (*χ*^2^ = 140.48, *p* < 0.001 and *χ*^2^ = 108.09, *p* = 0.006, respectively), but both solutions were rejected because one item (No. 16) had no salient loading (≥0.45), and two items (No. 7 and 9) did not load on any factor. These findings indicate no or insufficient contributions of these three items to any of those factors.

#### 3.1.2. Reliability

In addition to the results of the factor analysis of the K-ADAS (16 items), in the item-total correlation test, the coefficients for the three items (No. 7, 9, and 16) were less than 0.30, indicating that these three items did not contribute to this instrument (Table 2). Thus, based on the factor analysis and item-total correlation tests, we excluded items No. 7, 9, and 16 because of their insufficient contributions to this instrument. Then, we retested the validity and reliability of the K-ADAS (13 items).

### 3.2. Second Psychometric Properties of the Modified K-ADAS

#### 3.2.1. Construct Validity: Factor Analysis

After dropping the three items (No. 7, 9, and 16), the second EFA was conducted, and a three-factor solution, but not a four-factor solution, was selected, because it showed a better model fit than the four-factor solution based on CFI, TLI, and RMSEA tests. A three-factor structure model yielded good fit: CFI = 0.96, TLI = 0.92, SRMR = 0.04, and RMSEA = 0.08, with 90% CI = 0.04–0.11. Eigenvalues >1 for the unreduced correlation matrix were 5.22, 1.72, and 1.23. All 13 items loaded positively and significantly on one of the three factors, ranging from 0.45 to 0.85. Three items (No. 1, 2, and 3) had salient cross-loadings (≥0.45), and these items were assigned to the factor considering the level of loading coefficients and theoretical constructs (Table 3). Considering the three-structure model with 13 items, the factor inter-correlation was moderate to high (*r* = 0.32–0.92; all *p* < 0.05) (Table 4). We labeled the three factors as “Implications of Having an AD” (seven items: No. 5, 6, 8, 12, 13, 14, and 15); “Opportunity for Treatment Choices” (three items: No. 1, 2, and 3); and “Family Perspectives on ADs” (three items: No. 4, 10, and 11) (Table 3).

#### 3.2.2. Construct Validity: Known-Relationship Test

As hypothesized, significant correlations were found between the K-ADAS and perceived susceptibility (*r* = 0.37; *p* < 0.001) and severity (*r* = 0.30; *p* = 0.001), further supporting the construct validity of the K-ADAS (Table 5).

#### 3.2.3. Reliability

The reliability estimates for the three-factor model with 13 items was computed, and the reliability coefficient supported internal consistency reliability by an overall Cronbach’s alpha of 0.87, which improved from that of the 16-item K-ADAS (0.80). In item-to-total correlation analyses, all the correlation coefficients were >0.30, ranging from 0.41 to 0.74, supporting item homogeneity (Table 2). Cronbach’s alphas for the “Implications of Having an AD” subscale (seven items: No. 5, 6, 8, 12, 13, 14, and 15); “Opportunity for Treatment Choices” subscale (three items: No. 1, 2, and 3); and “Family Perspectives on ADs” subscale (three items: No. 4, 10, and 11) were 0.83, 0.85, and 0.74, indicating acceptable reliability (no table). In item-to-total correlation analyses, all the correlation coefficients in each subscale were >0.30, ranging from 0.47 to 0.80, supporting item homogeneity in each subscale.

## 4. Discussion

In this study, the psychometric properties of the K-ADAS were first evaluated, and the reliability and validity of the modified K-ADAS were supported by Cronbach’s alpha coefficients, item-to-total correlation coefficients, factor structures, and the significant relationships to perceived susceptibility and severity in community-dwelling Korean elders with chronic diseases. ADs have been exclusively considered in malignancy contexts, while few studies have examined their use in other populations in either non-malignancy or nonclinical settings in Korea. To facilitate their utility in the nonmalignant contexts, it is critical to assess people’s attitudes toward ADs using a reliable and valid instrument in nonmalignant settings. This is the first study that examined the psychometric properties of the K-ADAS to determine whether this instrument was reliable and valid to assess Korean elders’ attitudes toward ADs in a nonmalignant and nonclinical setting. 

Originally, a 17-item English version of the ADAS with four components was developed to assess the extent of positive or negative views about ADs and EOL decisions in medical inpatients with cardiac disorder (50%), gastrointestinal disorder (20%), or infection disease (12%) (mean age = 49 years). The developer revised the original 17-item ADAS to the modified version with 16 items. The full psychometric properties of the original version with 17 items or the revised versions with either 16 or 24 items have not been tested. Some Korean versions of the ADAS have been used among Koreans [32,33], but the full psychometric properties have not been tested in these either.

The full psychometric properties of the original and modified K-ADAS have been tested in this study for the first time. In the initial examination of the construct validity of the K-ADAS (16 items), a series of EFAs with three- to four-factor solutions provided significantly acceptable fits, but neither solution showed goodness of model fits [49]. Particularly, three items—a single item of the illness perception factor, No. 16 (I am not sick enough to have an AD), and two items of the impact of advance directives on the family factor, No. 7 and 9 (Having an AD would make my family feel left out of caring for me and Making my EOL treatment wishes clear with an AD would have no impact on my family)—did not demonstrate salient loadings. Furthermore, item-total correlation analyses showed that those three items did not contribute adequately to the scale (each of the coefficients < 0.30). Based on the findings from the initial factor and item-total correlation analyses, we eliminated those three items. A possible explanation for eliminating those items could be that public awareness of ADs has been meager in Korean elders in nonmalignancy contexts, because their use has been limited to terminal cancer patients. In this study, less than 10% of the participants knew about ADs. Thus, a lack of knowledge about ADs may cause the participants to be confused, making their responses ambiguous about whether they could be applied to any sickness or consider the effects on family.

The validity of the modified K-ADAS (13 items) was supported by the three-factor structures and the significant associations with perceived susceptibility and severity, as hypothesized. Except for reports about content validity and reliability, the validity of the English or Korean versions of the ADAS has not been tested in any populations. The developers suggested four theoretical components [31], but they have never been tested in any populations. Thus, this is the first study to examine the factor structures using a factor analysis and to further evaluate the construct validity using known relationship tests. The three-factor structure emerging from this study was “implications of having an AD”, “opportunity for treatment choices”, and “family perspectives on ADs.” This three-factor structure is somewhat similar to but also somewhat different from the four theoretical components (opportunity for treatment choices, impact of ADs on the family, effects of ADs on treatment, and illness perceptions (severity of their illness)) [31], with items in the family component in the original split into two components. The differences may be due to different cultures and settings. 

Further, inspection of this three-factor model showed that the remaining items in the original “impact of advance directives on the family” subscale were loaded onto the two factors; items of No. 5, 6, 8, and 12 were loaded on the subscale labeled “implications for having an AD” (No. 5, 6, 8, 12, 13, 14, and 15), and items of No. 10 and 11 were loaded on the subscale labeled “family perspectives on ADs” (No. 4, 10, and 11). One possible explanation for the double-loading of these items could be the vague boundary of patient autonomy regarding the healthcare decisions in the Korean health environment, which caused the items to be confusing to the participants for responses and, thus, resulted in unclear factor contributions to a family-specific substructure. In the Korean healthcare environment, particularly under Confucian belief, family–physician-shared decisions are respected more than patient autonomy regarding EOL care. Although patient autonomy perseveres, family members are largely surrogate decision-makers upon the provision of one’s palliative care and/or EOL medical care [51,52]. Under Confucian-rooted traditions, family members, particularly descendants, are actively engage in decision-making for patients’ EOL care [53,54], while patients—even those with terminal cancer—are often excluded from the loop of EOL discussion [55,56]. Older adults in this study may have perceived two questions as giving up on a patient or negligence in such a healthcare environment, leading to inconsistent item behavior. Thus, further studies are needed to verify the factor structures of the K-ADAS in different settings with different populations.

Reliability of the translated version (K-ADAS) in this study was first reported in low-income community-dwelling older adults with chronic illnesses. The internal consistency reliability based on Cronbach’s alphas in both the 16- and 13-item versions of the K-ADAS was acceptable, even though the alpha coefficient in the modified version (13 items) was slightly higher than that of the 16-item version (0.87 vs. 0.80). Previously, an original English version with 17 items [31] and its adapted version with 24 items regarding patients’ perceptions on ADs [57] showed desirable internal consistency, with Cronbach’s alpha coefficients of 0.74 and 0.86, respectively. In the two studies that investigated AD attitudes and factors influencing AD decisions in community-dwelling older adults [32] and elderly in- and outpatients with mainly solid malignancies [33], internal consistency was reported with a Cronbach’s alpha of 0.79 in both studies. The number of items impacts the Cronbach’s alpha, which increases with increases in the number of items [58]. In the initial psychometric test of the K-ADAS, the number of items was reduced from 16 to 13 items, but the Cronbach’s alpha increased from 0.80 to 0.87. This indicates that the modified K-ADAS has good internal consistency reliability with less burdens to participants. 

Considering the supported validity and reliability of the modified K-ADAS in this study, our findings initially provide insights into the AD attitudes in chronically ill people in the community. Assessment of AD attitudes using a reliable and valid measure, the modified K-ADAS, may facilitate a better understanding of and establish the nationwide use of ADs. In addition, this study has several unique contributions. The reliability and validity have been tested in nontypical populations of AD research. Despite the possible benefits of ADs [18,19] and the importance of attitudes toward ADs and ACP care in not only malignancy but also nonmalignancy settings, empirical evidence assessing attitudes toward ADs is lacking in nonmalignancy populations, particularly in a sample of elderly home residents [59]. To assess attitudes toward ADs in nonmalignancy populations, it is critical to use a reliable and valid instrument to assess them. In this study, the level of the attitudes toward ADs based on the original K-ADAS (16 items) among chronically ill older adults with low incomes was similar to those in other populations. The score of the original K-ADAS in this study was 46.49 out of 64, which was similar to the score of 45.12 out of 64 among the community-dwelling elderly Koreans recruited from senior centers [32]. The community-dwelling Korean elders seemingly had more positive attitudes than African American elders recruited from senior citizen centers, with their scores being 51.90 out of 96 on an adapted version of Nolan’s ADAS, while crudely transformed to 34.6 out of the 16-item max score of 64 [29]. Positive attitudes toward ADs were reported to be associated with a higher willingness to complete ADs [60]. This indicates that ADs may be introduced to community-dwelling Korean elders in advance. However, the scores in both Korean studies showing attitudes toward ADs among community-dwelling Korean elders and chronically ill Korean elders with low incomes can be improved further.

These findings derived from the community settings were consistent with evidence shown in patient populations, but more participants in clinical contexts indicated slightly more positive attitudes toward ADs [31,33,61]. For example, for AD attitudes of medical inpatients who stated that their illness status was serious, approximately two-thirds indicated their AD attitudes were moderately highly positive, with a mean score of 50.38 out of 68 [31]. Hospitalized patients also showed moderately highly positive attitudes toward ADs (66.9 out of 88), assessed using an adapted version of Nolan’s ADAS [57]. In the study, the majority of participants with completed ADs were White, female, and older and had lower educations and poor health perceptions. The majority of Hong Kong Chinese older adults with chronic diseases who have rarely heard of ADs or had such a discussion reported favorable attitudes toward ADs, and the majority were against futile life-sustaining treatment [61]. Older in- and outpatients with largely hepatobiliary and pancreatic cancers also showed moderately positive attitudes, with a mean score of 48.29 out of maximum score of 64 [33]. Prior and current evidence about moderately positive attitudes toward ADs indicates that, regardless of the illness status, earlier use of an AD is more likely feasible in participants from both public and clinical settings, with the majority believing in its use while healthy [29,31]. They believed that ADs would serve as a vehicle to facilitate the practice of patient autonomy while actively engaging in one’s care and assisting with informed and shared decision-making for EOL medical care and, further, ensuring their implementations as one’s wishes [29]. 

In Korea, attention to ADs has increased in both public and clinical contexts, and the increased attention is associated with growing patient autonomy for one’s own medical care and a pilot operation of the “Act on Decisions on Life-Sustaining Treatment” [14]. Negative AD attitudes were largely reported as one of the correlates of adverse impacts on health outcomes, including limited access to the underutilization of EOL discussions or ADs [13,29,45,62], while enhancing attitudes possibly contributed to increasing the completion of ADs [60]. We further investigated low-income elderly people’s attitudes in association with an individual’s perceived susceptibility and severity regarding EOL care, which showed significant correlations. Such a relationship was also shown in Korean American older adults [45]. These findings indicate that positive attitudes toward ADs may be needed to facilitate the discussion of EOL and ACP care in advance. A motivational stage-tailored ACP intervention proved beneficial in increasing knowledge, attitudes, self-efficacy, and perceived importance regarding ACP [63]. Future studies are warranted to identify modifiable factors, including AD attitudes, that contribute to good quality of care in futile medical care and to investigate whether enhancing these factors is effective for the optimal use of ADs and, further, ACP in various contexts.

### Limitations and Implications 

There were a couple of limitations in this study. Methodological issues, such as a relatively small sample and recruitment from one public health center, may limit study validity and generalizability. For an EFA of the 16-item K-ADAS, 80–160 cases were required. Despite at least 80 cases based on at least five times the variables for factor analysis [64] being met in this study, validation of the results is warranted. Homogeneity of the sample characteristics who were financially vulnerable and received a home visiting service and using only a single public health center limited the generalization of the study findings. Further studies are needed to examine the psychometric properties of the K-ADAS (16 items) and the modified K-ADAS (13 items) using larger samples as well as other patient populations in both clinical and nonclinical contexts and other stakeholders, including family members, social workers, chaplains, or policy-makers, whose perspectives and consensus are considered important for one’s EOL care.

Despite the limitations, our findings showing positive attitudes of lower-income chronically ill elderly people in the community have some implications for both research and practice. First, education and professional training on this kind of care involving both malignancies and nonmalignancies are needed to improve public awareness of, knowledge of, and attitudes toward ADs and reduce issues about ACP and preparedness of ADs among patients, families, and physicians. Such initiatives should begin as early as possible in public and clinical settings, particularly in primary care. Second, to fully utilize and adapt the K-ADAS to practice, more data-based research in various contexts is warranted. Families are important stakeholders in this care and surrogate decision-makers for critical medical treatment. Thus, further research needs to be done to examine family dynamics, family support, and their impacts on individual medical care regarding ADs or EOL. ACP has exclusively been used for terminally ill patients. However, earlier introduction of ACP to people, regardless of their conditions, is likely to be plausible, particularly by integrating it into primary care. In this process, any form of ADs can be introduced as a vehicle to facilitate one’s active engagement in one’s care and to improve communication for the continuity and quality of future EOL care. Third, investigations should also develop context-oriented communication models or systems that are better-suited to addressing EOL issues in a variety of the healthcare settings, yet empirical evidence for its use and potential outcomes is lacking. Given the existing hospice and nonhospice paradigms of palliative care largely in clinical settings, a public care model for ACP is particularly needed. Future research should target people in various contexts considering different groups of age, healthcare settings (e.g., malignancy and nonmalignancy), and quality of EOL care.

## 5. Conclusions

In this study, the reliability and validity of the modified K-ADAS were initially supported among community-dwelling elderly Koreans. Internal consistency reliability was supported by the adequate level of the Cronbach’s alpha; construct validity was supported by the factor structure and the significant associations with perceived susceptibility and severity. Thus, this modified K-ADAS can be used to assess AD attitudes of Koreans, specifically regarding the provision of treatment choices, impacts of ADs on the family and EOL treatment, and illness perceptions in the possession of ADs. Considering the differences in factor structures between the original four-factor structure in the English version and the modified K-ADAS, further studies may be needed to test the factor structures in different populations and settings.

## Figures and Tables

**Table 1:** Characteristics of older adults in the community (*N* = 118).

Characteristics		*n* (%)	Mean ± SD
Age (years), range (60–90)			75.09 ± 7.21
Gender	Female	98 (83.1)	
Marital Status	Married	46 (39.0)	
Education	Elementary or lower	85 (72.0)	
	Middle school	13 (11.0)	
	High school and higher	20 (16.9)	
Religion	(Yes)	78 (66.1)	
AD awareness	(Yes)	10 (8.5)	
Comorbidity, range (0–7)			1.47 ± 1.25
Chronic diseases *	Hypertension (Yes)	65 (55.1)	
	Diabetes (Yes)	47 (39.8)	
	Arthritis (Yes)	38 (32.2)	
	Gastric disorder (Yes)	38 (32.2)	
	Cardiovascular disease (Yes)	16 (13.6)	
	Cerebrovascular disease (Yes)	9 (7.6)	
	Dyslipidemia (Yes)	6 (5.1)	
	Others (Yes)	46 (39.0)	
Perceived susceptibility, range (10–35)			26.36 ± 6.34
Perceived severity, range (6–35)			25.55 ± 6.62
K-ADAS, range (32–60)			46.49 ± 5.24

Note. AD: advance directives and K-ADAS: Korean version of the Advance Directive Attitude Survey. * Self-reported. Others include asthma, benign prostatic hyperplasia, cystitis, dementia, kidney disease, or osteoporosis.

**Table 2:** Means of items and item-total correlations of the K-ADAS in older adults (*N* = 118).

Original Subscales	Items	Mean	SD	With 16 Items		With 13 Items	
				Item-Total Correlations	Alpha If Item Deleted	Item-Total Correlations	Alpha If Item Deleted
Opportunity for Treatment Choices	1. I have choices about the treatment I would receive at the end of my life.	3.05	0.68	0.48	0.78	0.53	0.86
	2. I would be given choices about the treatment I would receive at the end of my life.	2.97	0.67	0.44	0.78	0.52	0.86
	3. My doctor would include my concerns in decisions about my treatment at the end of my life.	2.97	0.63	0.42	0.79	0.46	0.87
	4. If I could not make decisions, my family would be given choices about the treatment I would receive.	3.08	0.60	0.38	0.79	0.41	0.87
Impact of Advance Directives on the Family	5. I think my family would want me to have an advance directive.	2.97	0.67	0.56	0.78	0.58	0.86
	6. Making my end of life treatment wishes clear with an AD would keep my family from disagreeing over what to do if I were very sick and unable to decide for myself.	2.99	0.58	0.45	0.78	0.45	0.87
	7. * Having an AD would make my family feel left out of caring for me.	2.66	0.73	−0.08	0.82	-	-
	8. Making my end of life treatment wishes clear with an advance directive would help to prevent guilt in my family.	2.84	0.61	0.45	0.78	0.48	0.87
	9. * Making my end of life treatment wishes clear with an AD would have no impact on my family.	2.62	0.67	0.15	0.80	-	-
	10. Having an AD would prevent costly medical expenses for my family.	3.07	0.75	0.54	0.78	0.54	0.86
	11. Having an AD would make sure that my family knows my treatment wishes.	2.99	0.62	0.64	0.77	0.67	0.86
	12. My family wants me to have an AD.	2.73	0.69	0.66	0.77	0.66	0.86
Effect of an Advance Directive on Treatment	13. Having an AD would make sure that I get the treatment at the end of my life that I do want.	2.91	0.60	0.74	0.76	0.74	0.85
	14. I trust one of my family or friends to make treatment decisions for me if I cannot make them myself.	3.02	0.60	0.48	0.78	0.51	0.86
	15. It is better to make an advance directive when you are healthy.	3.05	0.67	0.52	0.78	0.55	0.86
Illness Perception	16. * I am not sick enough to have an advance directive.	2.57	0.72	−0.21	0.83	-	-
	Total	2.91 *	0.66 *		0.80		0.87 *

Notes. * The items were reverse-coded. In the 13-item version, items No. 7, 9, and 16 were deleted because of no or insufficient contributions of the items to this instrument.

**Table 3:** Factor analysis with maximum likelihood (3-factor structure model) (*N* = 118).

Factors (Fs)	Items					Mean (± SD)		Factor 1	Factor 2	Factor 3
F1: Implications for Having an AD	5. I think my family would want me to have an advance directive.					2.97 (± 0.67)		0.72 *	−0.01	−0.08
	6. Making my end of life treatment wishes clear with an AD would keep my family from disagreeing over what to do if I were very sick and unable to decide for myself.					2.99 (± 0.58)		0.57 *	−0.14	−0.02
	8. Making my end of life treatment wishes clear with an advance directive would help to prevent guilt in my family.					2.84 (± 0.61)		0.52 *	0.10	−0.01
	12. My family wants me to have an AD.					2.73 (± 0.69)		0.72 *	−0.03	0.09
	13. Having an AD would make sure that I get the treatment at the end of my life that I do want.					2.91 (± 0.60)		0.85 *	0.04	0.02
	14. I trust one of my family or friends to make treatment decisions for me if I cannot make them myself.					3.02 (± 0.60)		0.45 *	0.01	0.17
	15. It is better to make an advance directive when you are healthy.					3.05 (± 0.67)		0.57 *	0.13	0.01
F2: Opportunity for Treatment Choices	1. I have choices about the treatment I would receive at the end of my life.					3.05 (± 0.68)		0.48 *	0.75 *	0.01
	2. I would be given choices about the treatment I would receive at the end of my life.					2.97 (± 0.67)		0.47 *	0.82 *	−0.01
	3. My doctor would include my concerns in decisions about my treatment at the end of my life.					2.97 (± 0.63)		−0.01	0.58 *	0.55 *
F3: Family Perspectives on ADs	4. If I could not make decisions, my family would be given choices about the treatment I would receive.					3.08 (± 0.60)		0.02	−0.04	0.61 *
	10. Having an AD would prevent costly medical expenses for my family.					3.07 (± 0.75)		0.31	−0.02	0.49 *
	11. Having an AD would make sure that my family knows my treatment wishes.					2.99 (± 0.62)		0.33	0.02	0.59 *
	Information criteria: Bayesian (BIC) = 2455.41					Eigen values		5.22	1.72	1.23
	Fit Indices from EFA Model	*χ* ^2^	*df*	*p*	CFI	TLI	SRMR	RMSEA	p (RMSEA < 0.05)	RMSEA 90% CI
		70.04	42	0.004	0.96	0.92	0.04	0.08	0.09	0.04, 0.11

Note. AD = advance directives, EOL = end-of-life, EFA = exploratory factor analysis, CFI = comparative fit index, TLI = Tucker-Lewis index, SRMR = standardized root mean square residual, RMSEA = root mean square error of approximation, and CI = confidence interval. * Significant at 0.05 level.

**Table 4:** Factor correlations of the Korean version of the Advance Directive Attitude Survey (*N* = 118).

Factors	K-ADAS_F1	K-ADAS_F2	K-ADAS_F3	K-ADAS_Total
	Pearson’s *r*			
K-ADAS_F1	1			
K-ADAS_F2	0.42 *	1		
K-ADAS_F3	0.32 *	0.60 *	1	
K-ADAS_Total	0.68 *	0.92 *	0.77 *	1

Note. K-ADAS: Korean version of the Advance Directive Attitude Survey; F1 = Implications for Having an AD; F2 = Opportunity for Treatment Choices; F3 = Family Perspectives on ADs; * <0.05.

**Table 5:** Associations of attitudes toward advance directives with illness perceptions (*N* = 118).

Illness Perception on EOL Experience	K-ADAS Total	
	*r*	*p*
Perceived susceptibility	0.37	<0.001
Perceived severity	0.30	0.001
